# Self-Healing of Recombinant Spider Silk Gel and Coating

**DOI:** 10.3390/polym15081855

**Published:** 2023-04-12

**Authors:** Shin-Da Wu, Wei-Tsung Chuang, Jo-Chen Ho, Hsuan-Chen Wu, Shan-hui Hsu

**Affiliations:** 1 Institute of Polymer Science and Engineering, National Taiwan University, Taipei 10617, Taiwan; 2 National Synchrotron Radiation Research Center (NSRRC), Hsinchu 30076, Taiwan; 3 Department of Biochemical Science and Technology, National Taiwan University, Taipei 10617, Taiwan; 4 Institute of Cellular and System Medicine, National Health Research Institutes, Miaoli 350, Taiwan

**Keywords:** recombinant spider silk, self-healing, β-sheet nanocrystal, in situ SAXS

## Abstract

Self-healing properties, originating from the natural healing process, are highly desirable for the fitness-enhancing functionality of biomimetic materials. Herein, we fabricated the biomimetic recombinant spider silk by genetic engineering, in which *Escherichia coli* (*E. coli*) was employed as a heterologous expression host. The self-assembled recombinant spider silk hydrogel was obtained through the dialysis process (purity > 85%). The recombinant spider silk hydrogel with a storage modulus of ~250 Pa demonstrated autonomous self-healing and high strain-sensitive properties (critical strain ~50%) at 25 °C. The in situ small-angle X-ray scattering (in situ SAXS) analyses revealed that the self-healing mechanism was associated with the stick-slip behavior of the β-sheet nanocrystals (each of ~2–4 nm) based on the slope variation (i.e., ~−0.4 at 100%/200% strains, and ~−0.9 at 1% strain) of SAXS curves in the high q-range. The self-healing phenomenon may occur through the rupture and reformation of the reversible hydrogen bonding within the β-sheet nanocrystals. Furthermore, the recombinant spider silk as a dry coating material demonstrated self-healing under humidity as well as cell affinity. The electrical conductivity of the dry silk coating was ~0.4 mS/m. Neural stem cells (NSCs) proliferated on the coated surface and showed a 2.3-fold number expansion after 3 days of culture. The biomimetic self-healing recombinant spider silk gel and thinly coated surface may have good potential in biomedical applications.

## 1. Introduction

Spiders are one of the oldest species on earth and have experienced evolution under various extreme conditions over hundreds of millions of years. Spider silks produced by spiders are unique protein-based biofibers possessing extraordinary properties, such as high tensile strength and toughness as well as supercontraction [1,2]. Meanwhile, spider silks are biocompatible and biodegradable [3]. These outstanding properties make spider silks an appealing natural material for use in the fields of medicine, textiles, and engineering [4]. However, the large-scale production of spider silks faces difficulty because spiders tend to kill each other when artificial breeding is carried out in a limited space. Meanwhile, the procedure of collecting spider silks from living spiders is cost-ineffective [5]. To overcome these problems, developing artificial recombinant spider silks by genetic engineering has become a promising solution for large-scale production [3,6]. For instance, *Escherichia coli* (*E. coli*), as a heterologous expression host, has widely been employed to produce recombinant spider silks via simple manipulation and cost-efficient production [7,8]. The produced recombinant spider silks can further self-assemble to form various products (e.g., hydrogel, films, microcapsules, tubes, and foams) for a wide range of applications [6].

Hydrogel, being a crosslinked three-dimensional (3D) polymeric network, is capable of retaining a large amount of water. Due to their high water content, hydrogels are appealing materials for use in biomedical applications [9]. Hydrogels based on recombinant spider silks can be formed from spider silk solutions by chemical or physical crosslinking [10]. Chemically crosslinked spider silk hydrogel involving the use of toxic crosslinkers, as well as initiators or catalysts in the crosslinking reaction, may be less favorable for biomedical applications [11,12]. In comparison, physically crosslinked spider silk hydrogel formed through the self-assembly process has the advantages of simple production as well as no need for additional toxic elements (e.g., crosslinkers, initiators, or catalysts) [12,13]. The mechanism of the self-assembly process is concentration-dependent gelation via nucleation and aggregation [12,14,15]. Upon gelation to a hydrogel, the spider silk is arranged into physically crosslinked β-sheet nanocrystal structures by chain entanglements and hydrophobic interactions [15,16,17]. The fabricated physically crosslinked recombinant spider silk hydrogel with semiflexible nature has a similar polymeric network to that of other biopolymers, which provides great potential for various applications [11,18,19].

Self-healing properties, originating from the natural healing process of all multicellular organisms, have received considerable attention in recent years due to the ability to heal structural damages and recover the original functions [20]. For instance, the natural spider silk fibers showed an intrinsic self-healing property, which is beneficial to restoring the morphology of spider webs after repeated damage [21]. Inspired by this fitness-enhancing functionality that has evolved under various surroundings for hundreds of millions of years, researchers have tried to develop materials with biomimetic self-healing properties [20]. The biomimetic self-healing materials are mainly classified into two categories based on the type of reversible interaction: dynamic supramolecular interactions and dynamic covalent bond interactions [22]. The dynamic supramolecular interactions have a better self-healing performance than the dynamic covalent bond interactions because of the lower bond energy [23]. Hydrogen bonding, as one of the common dynamic supramolecular interactions, has the advantages of natural reversibility as well as fast healing speed and high healing efficiency [20,24]. Due to these merits, Koga et al. developed a biomimetic spider silk-inspired film with a self-healing property based on multiple reversible hydrogen bonding [25]. However, the fabrication involved the use of toxic organic solvents, which may limit the development of biomedical applications owing to the residual solvents [26]. These things considered, to the best of our knowledge, the self-healing property and the healing mechanism of the recombinant spider silk hydrogel have not been reported.

In this study, we develop the biomimetic recombinant spider silk through genetic engineering. The biomimetic recombinant spider silk was then self-assembled into the physically crosslinked hydrogel. The recombinant spider silk hydrogel showed autonomic and reversible self-healing behavior. The possible self-healing mechanism of the hydrogel examined by in situ small-angle X-ray scattering (in situ SAXS) was identified as the reversible hydrogen bonding within the β-sheet nanocrystal. The recombinant spider silk cast coated and dried from the hydrogel demonstrated a self-healing capacity and cytocompatibility. The fabricated biomimetic self-healing recombinant spider silk hydrogel and surface coating may have good potential in terms of biomedical applications.

## 2. Materials and Methods

### 2.1. Production of Recombinant Spider Silk: Gene Construction and Protein Expression

The type 2 major ampullate silk spidroin-associated gene from the spider *Nephila pilipes* was identified by Sanger and next-generation sequencing (Illumina NovaSeq 6000; Illumina, San Diego, CA, USA). Specifically, the signature repetitive unit, containing alanine-rich and proline-rich motifs, was cloned by the Biobrick restriction enzyme assembly strategy (NheI and SpeI enzyme sets; New England BioLabs, Ipswich, MA, USA) to generate a 32-repeated synthetic spidroin gene. Subsequently, the spidroin gene was flanked by N-terminal and C-terminal domains, and the resulting constructed spidroin was cloned in the pET28 expression vector and transformed into *E. coli* BLR(DE3) cells for recombinant spider silk protein expression via 0.25 mM isopropyl β-D-1-thiogalactopyranoside (IPTG) induction at 20 °C for 16–20 h.

### 2.2. Purification of Recombinant Spider Silk Protein

BLR(DE3) cell pellets expressing the recombinant spider silk were responded in lysis buffer (10 mM Tris-HCl at pH 8.0, 1.25 mg/mL lysozyme, and 0.5% *v/v* Triton X-100) and subjected to ultrasonication for cell lysis on ice (450 W, on/off pulse cycle of 3 s/5 s for a total of 120 min). Subsequently, the samples were centrifuged and resuspended with a 5% SDS buffer (10 mM Tris-HCl at pH 8.0) with sonication (20.334 kHz) for a total of 20 min on ice, followed by four cycles of centrifugation and rinsing with deionized water. After the removal of impurities, the resultant white pellet of silk protein was frozen with liquid nitrogen and lyophilized by a vacuum freeze dryer (under a pressure of 1 Pa at −20 °C). The final silk powder was kept in the desiccator at room temperature for storage.

### 2.3. Protein Analysis of Recombinant Spider Silk

For protein analysis, SDS-polyacrylamide gel electrophoresis (SDS-PAGE) was performed. Briefly, the dried silk was dissolved in hexafluoro-2-propanol (HFIP; Sigma-Aldrich, St. Louis, MI, USA), mixed with lithium dodecyl sulfate (LDS) sampling buffer, and heated at 95 °C for 5–10 min. Subsequently, the silk samples were loaded to an 8% SDS-polyacrylamide gel and analyzed by electrophoresis using 3-(N-morpholino) propane sulfonic acid (MOPS) running buffer. Afterward, the gel was removed and stained with Commissive Blue R-250 for 20–30 min and then destained with a destaining solution for further gel imaging.

### 2.4. Fabrication of Recombinant Spider Silk Hydrogel and Coated Surface

For hydrogel fabrication, the recombinant spider silk powder was first dissolved in 6 M guanidinium thiocyanate solution at the silk concentration of 8% and subsequently transferred into a dialysis tube (12–14 kDa cutoff membranes). The silk-containing tube was then dialyzed against fresh 10 mM Tris-HCl buffer at 25 °C, with the duration of the first two cycles being 3 h and a subsequent cycle continuing for 16 h. The dialyzed silk products were then kept at 4 °C for 4 days to obtain stable silk hydrogels. For the fabrication of surface coating, the recombinant spider silk hydrogel was placed onto a plastic petri dish (Greiner). After drying for 24 h at the ambient temperature (25 °C), the dry coating of recombinant spider silk was obtained.

### 2.5. FT-IR Analysis of Recombinant Spider Silk Hydrogel

The secondary structure analysis of spider silk hydrogel was carried out using the attenuated total reflectance-Fourier transform infrared (ATR-FTIR). Briefly, the spider silk hydrogel samples were submerged into liquid nitrogen and lyophilized by a vacuum freeze dryer. Afterward, the ATR-FTIR spectrum of the sample was detected using an FTIR spectrophotometer (Spectrum 100 model, PerkinElmer, Waltham, MA, USA) with a setup of the ATR mode. The sample was scanned 32 times with a resolution of 0.5 cm^−1^ in the wavelength range of 600–4000 cm^−1^. The resulting amide I area of the ATR-FTIR spectrum (wavenumber between 1580 and 1720 cm^−1^) was chosen for Gaussian deconvolution via Origin 9.0 (OriginLab, Northampton, MA, USA), followed by the subsequent identification of secondary structures.

### 2.6. Self-Healing Properties of Recombinant Spider Silk Hydrogel and Coated Films

To demonstrate the self-healing property of recombinant spider silk hydrogel, the hydrogel was cut into two halves by a sharp blade and then brought into physical contact in ambient air (25 °C) for 12 h without external intervention to test if they formed an integrated unit. To demonstrate the self-healing property of the recombinant spider silk coating, the surface of the dried gel coating was first gently scratched by a sharp blade. Subsequently, the scratched surface was immersed in deionized water for 10 min and then dried in ambient air (25 °C) for 6 h. The self-healing property of the coated films was evaluated by an observation of the scratch variation.

### 2.7. Rheological Properties of Recombinant Spider Silk Hydrogel

For rheological evaluation, the dynamic behavior of the recombinant spider silk hydrogel was measured by a rheometer (HR-2, TA Instrument, New Castle, DE, USA) with a cone plate geometry. The diameter of the upper cone was 40 mm with a 2° angle. The measurements were conducted in three different modes at 25 °C. For a time-dependent measurement, the shear moduli [i.e., storage modulus (G′) and loss modulus (G″)] of the hydrogel were measured against time with an oscillatory strain of 1% and a frequency of 1 Hz. For a strain-dependent measurement, the shear moduli of the hydrogel were measured against strains (1–500%) at a frequency of 1 Hz to determine the critical strain where a gel-to-sol transition occurred. For evaluating the self-healing property, the hydrogel was measured through continuous step changes of the oscillatory strain (damaging-healing cycles) at a frequency of 1 Hz to test the strain-induced structure destruction and recovery. Structure destruction was induced at a strain (60%) larger than the critical strain for a period of 5 min, and structure recovery was evaluated by decreasing the strain to the initial low strain (1%) for the same period of time (5 min).

### 2.8. In Situ SAXS Analysis of Recombinant Spider Silk Hydrogel

To analyze the structural variation of self-healing recombinant spider silk hydrogel under different strains, small-angle X-ray scattering (SAXS), combined with an in situ rheometer (Physica MCR-501, Anton Paar, Graz, Austria), was conducted at the beamline station 25A1 of Taiwan Photon Source (TPS 25A1) at National Synchrotron Radiation Research Center (NSRRC), Hsinchu, Taiwan. The SAXS profiles were measured under different oscillatory strains [i.e., 1% (beginning), 100%, 200%, and 1% (after 200%)] at 25 °C and a frequency of 1 Hz.

### 2.9. Cell Culture on Recombinant Spider Silk Substrate

Mouse neural stem cells (NSCs) were used for the cell culture test. NSCs derived from the brains of adult mice were cultured in Ham’s F-12 and HG-DMEM (1:1), with 10% fetal bovine serum (FBS; Caisson), 1% penicillin-streptomycin-amphotericin (PSA; Caisson), and 400 mg/mL G418 (Invitrogen), in a humid incubator containing 5% CO_2_ at 37 °C. The culture medium was refreshed every day. The recombinant spider silk-coated surface (area of 1.9 cm^2^), in a 24-well plate, was immersed in 75% ethanol for 1 h and irradiated with UV light for 1 h before NSC inoculation (cell density 4 × 10^4^ cells/cm^2^). The proliferation of cells on the surface was evaluated by the Cell Counting Kit-8 (CCK-8; Sigma-Aldrich) assay [27]. The reacted CCK-8 solution was mixed gently to ensure uniformity and then collected into a 96-well plate. The absorbance, at a wavelength of 450 nm, was detected by a plate reader (SpectraMax M5, Molecular Devices, San Jose, CA, USA). Statistical differences between the experimental groups were performed with the student’s *t*-test. The result was considered statistically significant when the *p*-value was smaller than 0.05.

### 2.10. Conductivity of the Recombinant Spider Silk Substrate

The electrical conductivity of the recombinant spider silk substrate was measured using a single-channel system sourcemeter (2601B, Keithley Instruments, Inc., Cleveland, OH, USA) under an applied voltage of 1 V at 25 °C.

## 3. Results

### 3.1. Fabrication of Recombinant Spider Silk

**Figure 1:**
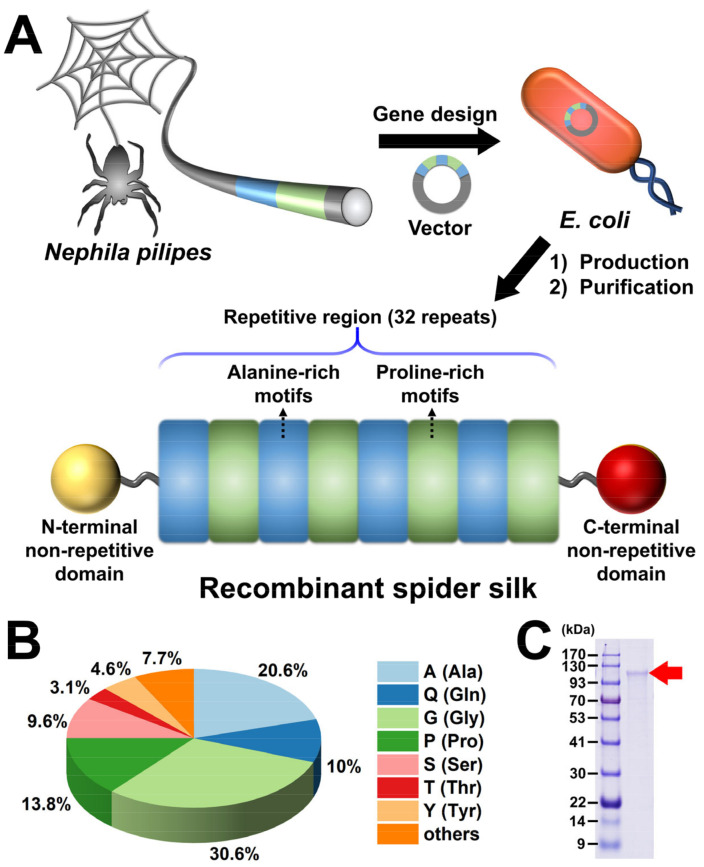
Schematic illustration of design for the recombinant spider silk. (**A**) Production and the modular structure of recombinant spider silk. (**B**) Amino acid sequence ratio of the recombinant spider silk. (**C**) The protein size of the recombinant spider silk, visualized by SDS-polyacrylamide gel electrophoresis (SDS-PAGE).

The recombinant spider silk was designed and generated via a genetic engineering approach, as shown in Figure 1A. First, the spidroin gene from the spider *Nephila pilipes* was synthesized. Subsequently, the spidroin gene was cloned in the vector and transformed into *E. coli* for recombinant spider silk protein expression. After the production and purification process, the final recombinant spider silk product containing N-terminal and C-terminal domains, as well as the tandem repetitive region in the presence of alanine-rich and proline-rich motifs, was obtained. The amino acid sequence ratio of the recombinant spider silk was analyzed, as shown in Figure 1B. The representative amino acid sequences were Gly and Ala as well as Pro, indicating the low sequence complexity of the recombinant spider silk. The molecular weight of the recombinant spider silk protein detected by SDS-PAGE was ~120 kDa, as shown in Figure 1C. Further image analysis of spider silk protein extracted from SDS-PAGE data using ImageJ (version 1.52a, National Institutes of Health, Bethesda, MD, USA) demonstrated an overall purity of more than 85%.

### 3.2. Fabrication of Recombinant Spider Silk Hydrogel

**Figure 2:**
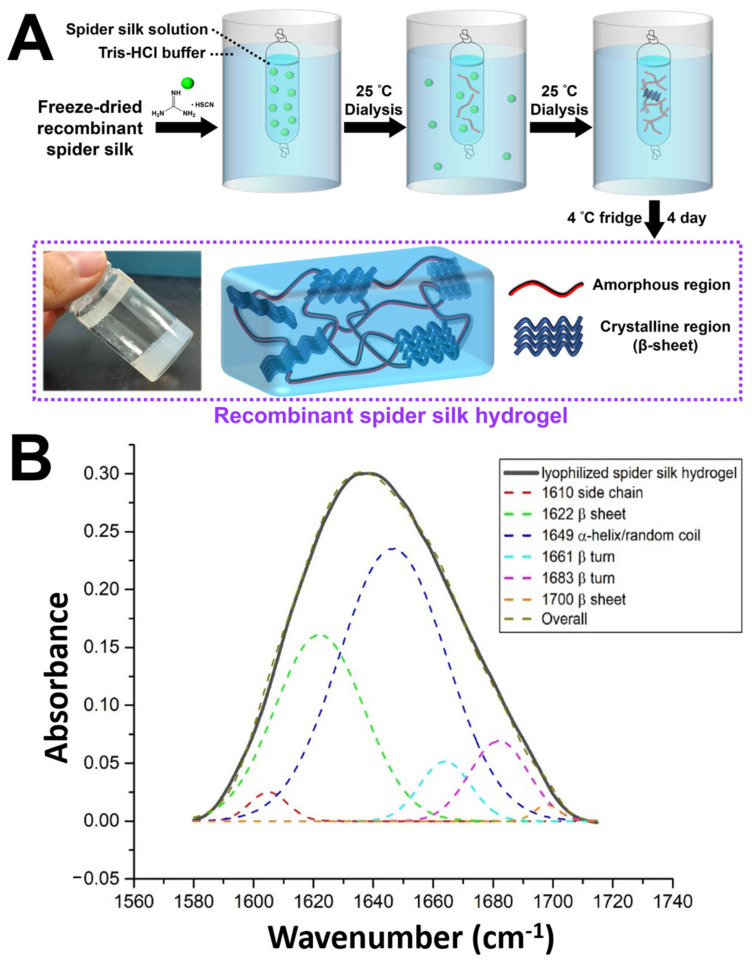
Fabrication and characterization of the recombinant spider silk hydrogel. (**A**) Schematic illustration of the fabrication procedure of the recombinant spider silk hydrogel. The inset shows the image of the fabricated recombinant spider silk hydrogel. (**B**) The attenuated total reflectance-Fourier transform infrared (ATR-FTIR) deconvolution analysis of lyophilized spider silk hydrogel.

The fabrication procedure of the recombinant spider silk hydrogel is shown in Figure 2A. First, the freeze-dried recombinant spider silk powder was denatured in guanidinium thiocyanate solution to obtain the recombinant spider silk solution. The silk solution was then dialyzed against the Tris-HCl buffer for two days at 25 °C. During dialysis, the silk solution spontaneously self-assembled into hydrogel inside the dialysis tube. The stable physically crosslinked recombinant spider silk hydrogel was obtained after storage of the self-assembled hydrogel in a 4 °C fridge for 4 days.

### 3.3. FTIR Analysis of Recombinant Spider Silk Hydrogel

In spider silk structural research, the amide I region, generally between 1600 and 1700 cm^−1^, has been widely exploited as a hallmark for estimating secondary structures [28]. According to Figure 2B, a series of peak assignments and fitting was carried out, with the β-sheet associated peak designated at 1622 cm^−1^, random coil at 1649 cm^−1^, and β-turn peaks at 1661 and 1683 cm^−1^ [29]. The relative content of the β-sheet structure over the total signals was estimated at around 30.9%, as an index for the crystallinity of the silk sample.

### 3.4. Self-Healing Property of Recombinant Spider Silk Hydrogel

**Figure 3:**
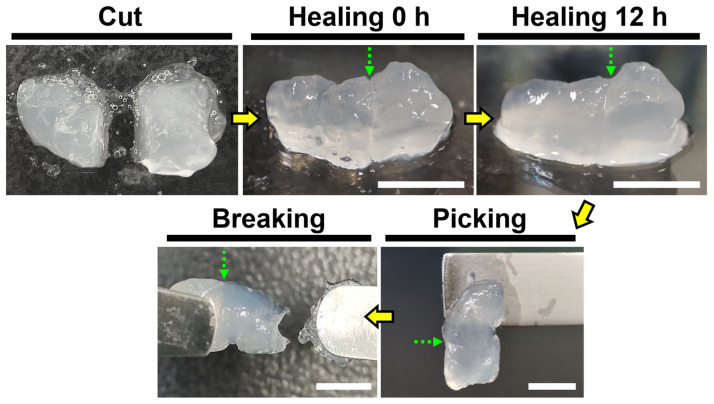
The self-healing behavior of the recombinant spider silk hydrogel, demonstrated by the gross test at 25 °C. The green dotted arrows show the cut section of the hydrogel. Scale bars represent 5 mm.

The macroscopic self-healing behavior of the recombinant spider silk hydrogel is demonstrated in Figure 3. The hydrogel was cut into two halves and then brought into physical contact at 25 °C. After 12 h, the two hydrogel halves healed automatically and showed a smoother appearance. To verify the healing of the two hydrogel halves, the integrated unit was picked up vertically fromhalf of it. The image showed that one half of the integrated unit could support the weight of another half without breaking. In addition, the integrated unit was stretched until breaking with a pair of tweezers. The location where the breaking occurred was different from the self-healed site of the hydrogel, verifying the self-healing ability of the recombinant spider silk hydrogel.

### 3.5. Rheological Properties of Self-Healing Recombinant Spider Silk Hydrogel

**Figure 4:**
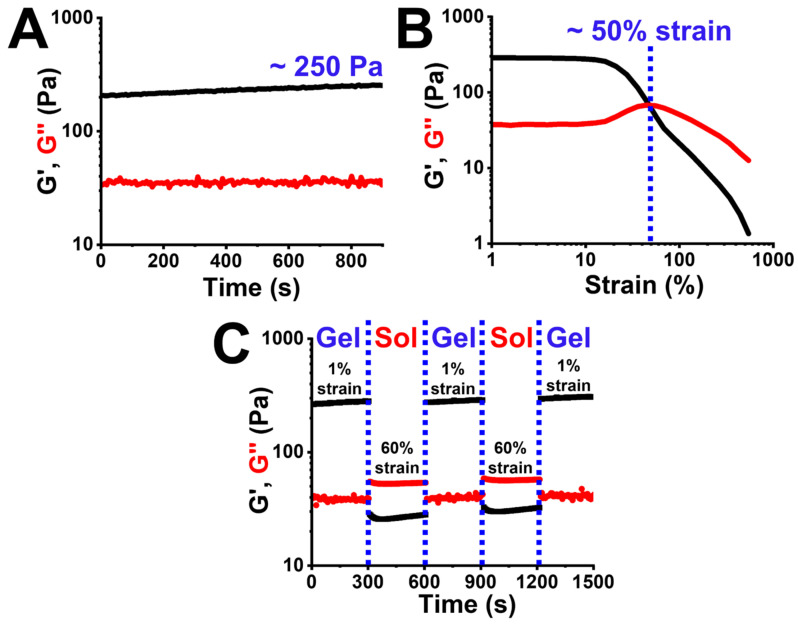
Rheological properties of the self-healing recombinant spider silk hydrogel at 25 °C. (**A**) The time-dependent moduli (G′ and G″) of the hydrogel were measured at 1% strain and 1 Hz. (**B**) The strain-dependent moduli (G′ and G″) were measured at 1 Hz. (**C**) The damaging-healing property of the self-healing recombinant spider silk hydrogel was measured through the continuous step changes of oscillatory strain at 1 Hz.

The rheological properties of the recombinant spider silk hydrogel are shown in Figure 4. For time-dependent measurement, the G′ value of the hydrogel at equilibrium was ∼250 Pa (Figure 4A). The critical strain for gel-to-sol transition (structure destruction) was determined by a strain-dependent measurement. The result showed that the critical strain of the hydrogel was ~50% (Figure 4B). The self-healing property of the recombinant spider silk hydrogel was evaluated by continuous step changes of the oscillatory strain between a lower strain (1%) and a higher strain (60%) that exceeded the critical strain (Figure 4C). At the lower strain (1%), G′ was higher than G″ and both shear moduli kept steady with time. Regarding the higher strain (60%), the hydrogel showed G′-G″ crossover and turned into the sol state. When reversing to the lower strain (1%), both shear moduli (G′ and G″) of the hydrogel recovered immediately to their initial values. The reproducible rheological property identified during multiple damaging-healing cycles confirmed the self-healing behavior of the recombinant spider silk hydrogel.

### 3.6. In Situ SAXS Analysis of Self-Healing Recombinant Spider Silk Hydrogel

**Figure 5:**
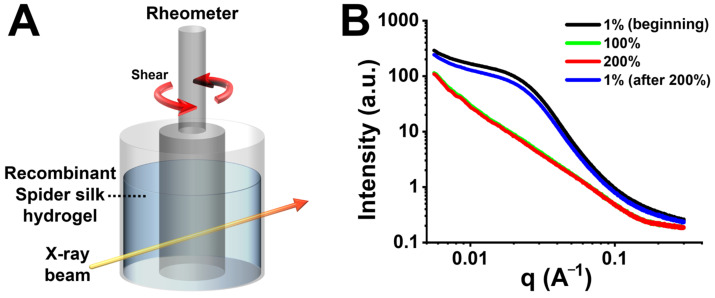
In situ small-angle X-ray scattering (in situ SAXS) measurements of the self-healing recombinant spider silk hydrogel at 25 °C. (**A**) Schematic illustration of the experimental setup for in situ SAXS measurements. (**B**) The small-angle X-ray scattering (SAXS) profiles of the recombinant spider silk hydrogel under different strains [i.e., 1% (beginning), 100%, 200%, and 1% (after 200%)].

The self-healing property of the recombinant spider silk hydrogel was further supported by in situ SAXS analyses, as shown in Figure 5. The in situ SAXS setup combined the collimated X-ray beam with the rheometer that loaded with the recombinant spider silk hydrogel (Figure 5A). The SAXS profiles were measured at different oscillatory strains to analyze the structural variation of the hydrogel (Figure 5B). At the initial low strain [i.e., 1% (beginning)], the SAXS profile revealed a broad hump signal in the q-range of ~0.008–0.08 Å^−1^, which may reflect the featured spherical aggregates [30,31]. The radius of the spherical aggregates was calculated to be ~9 nm. The distance among the spherical aggregates was ~8–80 nm. The slope of the curve in the high q-range (i.e., 0.15–0.3 Å^−1^) was ~−0.9, which may be ascribed to the rod-like β-sheet nanocrystals [32]. The size of the β-sheet nanocrystals was ~2–4 nm. Upon applying the high strains (i.e., 100% and 200%) that exceeded the critical strain, the hump disappeared in the SAXS profiles. Such variation indicated that the spherical aggregates were destroyed. The fractal dimension calculated based on the power law and the slope of the curve was ~1.7, which suggested an irregular shape of the aggregates. The slope of the curve in the high q-range decreased to ~−0.4, indicating the failure of the β-sheet nanocrystals. Afterward, when the strain was reversed from 200% to the initial low value of 1%, the hump reappeared at the same q region with a similar intensity to that of the initial SAXS profile. This finding indicated the structure recovery of the spherical aggregates. Furthermore, the slope of the curve in the high q-range returned to a range near the initial value (i.e., ~−0.9), indicating the repairing of the rod-like β-sheet nanocrystals.

### 3.7. Self-Healing Mechanism of Recombinant Spider Silk Hydrogel

**Figure 6:**
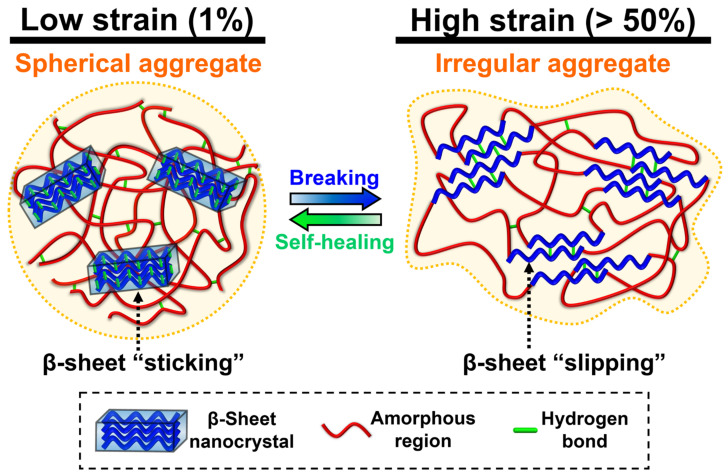
The hypothetical self-healing mechanism of the recombinant spider silk under low strain (1%) and high strain (>50%).

The possible mechanism for the autonomic self-healing property of the recombinant spider silk hydrogel is illustrated in Figure 6. The recombinant spider silk consisted of segmented amino acid sequences with alanine-rich and proline-rich motifs (cf. Figure 1A). The alanine-rich and proline-rich motifs were driven to form rod-like β-sheet nanocrystals (each of ~2–4 nm) and amorphous regions, respectively, within the hydrogel. At the lower strain (1%), the hydrogel was stable because the β-sheet nanocrystals were firmly locked by the hydrogen bonds. Meanwhile, some hydrogen bonds also existed within the amorphous regions. A large amount of hydrogen bonding allowed the formation of spherical aggregates (each of ~9 nm). Upon applying a higher strain that exceeded the critical strain (>50%), the spherical aggregates were destroyed and became irregular aggregates (cf. Figure 5B). Such structural variation could be mainly ascribed to the rupture of hydrogen bonds within the β-sheet nanocrystals, leading to the slipping and failure of the β-sheet nanocrystals. Meanwhile, the hydrogen bonds within the amorphous regions were also broken. Upon reversing to the lower strain (1%), the spherical aggregates reformed. This structure recovery could be mainly attributed to the regaining of the hydrogen bonds within the β-sheet nanocrystals, leading to the sticking of the β-sheet nanocrystals. The stick-slip behavior of the β-sheet nanocrystals enabled the damaging and healing of the hydrogel network. In addition, some hydrogen bonds within the elastic amorphous regions may also reform and facilitate the healing of hydrogel.

### 3.8. Fabrication and Characterization of Recombinant Spider Silk-Coated Substrate

**Figure 7:**
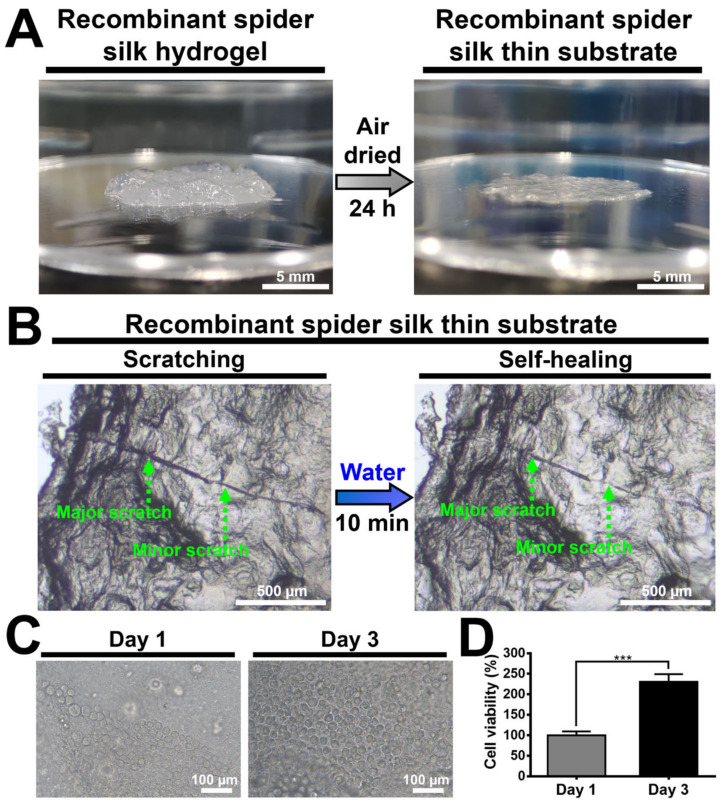
Fabrication, self-healing, and cytocompatibility of the recombinant spider-silk-coated films. (**A**) Images showing the recombinant spider silk fabricated from casting the recombinant spider silk hydrogel under ambient air and drying for 24 h at 25 °C. The thickness of the film was ~200 µm. (**B**) Optical microscopic images showing the healing of scratches on the surface of the recombinant spider silk after immersion in water for 10 min and then drying in ambient air for 6 h at 25 °C. The green dotted arrows show the major scratches and the minor scratches. The proliferation of neural stem cells (NSCs) on the recombinant spider silk substrate was evaluated by (**C**) observation under the optical microscope and (**D**) the Cell Counting Kit-8 (CCK-8) assay. The cell viability was deducted from that of the control group (same substrates but without cells) and normalized to the initial value (i.e., cell viability at 1 day serving as 100%). *** *p* < 0.001 between the indicated groups.

The recombinant spider silk substrate was fabricated by coating and drying the recombinant spider silk hydrogel at ambient air for 24 h, as shown in Figure 7A. The thickness of the dried silk substrate (thin film) was about 200 µm. The electrical conductivity of the dried silk substrate was ~0.4 mS/m. The self-healing behavior of the recombinant spider silk thin substrate demonstrated by a scratch test is shown in Figure 7B. After the scratched surface was treated with water for 10 min and dried for 6 h, the major scratch width and the minor scratch width on the surface of the substrate were reduced from 23.2 ± 2.5 to 15.1 ± 3.6 µm and from 14.2 ± 1.9 to 9.9 ± 1.3 µm, respectively. Meanwhile, the appearance of both the major and minor scratches after the treatment became less distinct than that of the initial scratches. These scratch changes verified the healing behavior of the recombinant spider silk. The cytocompatibility of the recombinant spider silk substrate is demonstrated in Figure 7C,D. The proliferation of NSCs on the spider silk substrate during a period of 3 days was observable. After 3 days of cell culture, the cell viability significantly increased to ~230%.

## 4. Discussion

Major ampullate spidroin (MaSp), also known as spider dragline silk, receives more attention than other spidroins due to its known protein sequence and desired mechanical properties [33]. MaSp includes two main types of proteins, i.e., major ampullate spidroin 1 (MaSp1) and major ampullate spidroin 2 (MaSp2). The most apparent difference between MaSp1 and MaSp2 is the proline content in the protein sequence. MaSp2 is a proline-rich protein (~9%), whereas MaSp1 contains few proline residues (<1%) [34,35]. The proline-rich motif in MaSp2 acts to form the β-turn spiral structure for providing high elasticity [36], which plays a key role in the function of the natural spider silk, such as supercontraction behavior [4,37]. For the large-scale fabrication of the biomimetic MaSp2 spider silk, various expression systems (e.g., bacteria, yeasts, plants, insects, silkworms, and animals) are used to attempt the recombinant production of spider silk [38]. *E. coli* is one of the most popular expression platforms for the large-scale genetic engineering production of recombinant spider silk due to the advantages of its well-known genetics, easy genetic manipulation, short life cycle, and easy culture [35,39]. In this study, *E. coli* was employed as a heterologous expression host to synthesize the recombinant MaSp2 spider silk.

The protein sequence of the designed recombinant MaSp2 spider silk based on the spider *Nephila pilipes* has a highly repetitive core region consisting of alternating alanine-rich and proline-rich motifs. The alanine-rich motifs dominated by A_n_ or (GA)_n_ (A: Ala; G: Gly) form the β-sheet crystalline region for the high tensile strength of the spider silk, while the proline-rich motifs dominated by GPGXX (P: Pro; X: Gly, Gln, Tyr, Ala, Ser) form the amorphous region for the extensibility of the spider silk [40,41]. The two non-repetitive terminal domains (i.e., N-terminal domain and C-terminal domain) flanking the repetitive core region are important for the storage of proteins in the gland and the initiation of fiber assembly [42,43,44]. The synthesized recombinant MaSp2 spider silk powder was further processed to the recombinant spider silk hydrogel according to a previously published procedure [12]. The self-assembling mechanism of the recombinant spider silk hydrogel during dialysis is the nucleation-aggregation process followed by concentration-dependent gelation [11,14]. During gelation, the α-helical and random coil conformations of the spider silk protein chains are arranged into β-sheet conformations due to the hydrophobic interactions and entanglements [12,15,16,17]. The fabricated recombinant spider silk hydrogel in the present study involving an aqueous solution could possess greater potential for broader biomedical applications, compared to other recombinant spider silk gels derived from organic solvents [13].

The fabricated recombinant spider silk hydrogel evaluated by the rheological measurements was a soft gel (G′ ~250 Pa) [45]. The critical strain of the recombinant spider silk hydrogel was low (~50%), indicating the high strain-sensitive property. The high strain-sensitive property is typically attributed to the supramolecular interaction of the physical hydrogel, e.g., hydrogen bonding [46,47,48]. The bond energy of hydrogen bonding within the physical hydrogel is much lower than that of covalent bonding within the chemical hydrogel [49,50]. Therefore, when applying a higher strain that exceeded the critical strain, the weak hydrogen bonding was easily broken, and the 3D network of the physical hydrogel was then destroyed into a sol-like state [51]. However, due to the reversible nature of hydrogen bonding, many physical hydrogels in the published literature could reconstruct their network (i.e., self-healing) after reversing to the initial low strain [52]. In this study, the self-healing behavior of the recombinant spider silk hydrogel was observed and confirmed by the gross test and the rheological damaging-healing test. It has to be noted that, the rheological damaging-healing test was conducted right after the strain-dependent measurement. Due to the large applied strain (i.e., 500%) in the strain-dependent measurement, the hydrogel was broken thoroughly and needed more time to repair. Therefore, within the rheological damaging-healing test, the initial shear moduli were slightly lower than the final shear moduli of the self-healed hydrogel. This result also confirmed the self-healing property of the recombinant spider silk hydrogel.

Self-healing materials can be categorized into extrinsic healing and intrinsic healing, or non-autonomous healing and autonomous healing [53]. The extrinsic self-healing materials employ the encapsulation of external healing agents to achieve self-healing. However, the major disadvantage of extrinsic healing is the limited one-time healing cycle [54]. In comparison, the intrinsic self-healing materials employing reversible bonds to restore the structure can heal multiple cycles without a need for healing agents or catalysts. The non-autonomous healing materials require external stimuli, such as light and heat, to trigger structural recovery. However, external stimuli could have adverse effects on the cells in biomedical applications [55]. In comparison, autonomous healing materials can automatically and reversibly repair the damages [53]. In this study, the self-healing property of the recombinant spider silk hydrogel is autonomous and intrinsic, according to the gross test and the rheological damaging-healing test.

The β-sheet nanocrystals are one of the key elements for the toughness and strength of natural silks (e.g., spider silk and silkworm silk) [30,56]. The sizes of the β-sheet nanocrystals in natural silks are typically less than 10 nm in all three dimensions [57]. According to the molecular dynamics simulations, Keten et al. demonstrated that the β-sheet nanocrystals with different sizes within the spider silk can be categorized into two types [58]. The critical size of the β-sheet nanocrystals was 2–4 nm. During large lateral loading, the failure mechanism for the β-sheet nanocrystal with a size larger than the critical value was dominated by the bending deformation [58]. The cracks caused by the non-uniform tension were easily attacked by water molecules in the surroundings, which resulted in a large-scale rupture of the hydrogen bonds [59]. In comparison, the failure mechanism for the β-sheet nanocrystal of the smaller size was dominated by the uniform shear deformation, owing to the greater stiffness and fracture resistance [58]. Under a homogeneous shear, the cooperative rupture of the hydrogen bonds prevented the internal defects of the nanocrystals from being exposed to the surrounding water. Based on the characteristic stick-slip mechanism of the hydrogen bonds via repeatable rupturing and reformation, the self-healing behavior of the smaller β-sheet nanocrystal could occur. In this study, the failure mechanism for the β-sheet nanocrystal of the smaller size (~2–4 nm) may be dominated by the uniform shear deformation. Through the stick-slip mechanism of the β-sheet nanocrystals under low and high shear deformation, the self-healing behavior of the recombinant spider silk hydrogel was achieved.

The recombinant squid ring teeth (SRT) protein, which consisted of highly repetitive tandem polypeptides similar to the recombinant spider silk protein, also showed the self-healing behavior discussed in the published literature [60,61,62]. For instance, Ding et al. showed that the autonomous self-healing behavior of the SRT protein hydrogel was based on the breakage and reformation of the β-sheet nanocrystals [60]. For example, Pena-Francesch et al. fabricated the SRT protein film with the self-healing property, which mainly relied on the reversible hydrogen bonding within the β-sheet nanocrystals [61]. In the film system, water was necessarily employed for breaking the hydrogen bonding within the amorphous region, which can facilitate the chain mobility and network repair [4,61,63]. Based on the same principle and similar conditions, Koga et al. demonstrated that the spider-silk-inspired multiblock copolymer film showed the self-healing behavior, which also relied on the reversible hydrogen bonding within the β-sheet nanocrystals [25]. In this study, we infer that the self-healing mechanism of the recombinant spider silk thin surface coating was based on the chain diffusion within the amorphous region and the reversible hydrogen bonds within the β-sheet nanocrystals.

The advantage of our recombinant spider silk-coated film is the feasibility of biomedical applications due to no involvement of organic solvents in the fabrication process [26]. NSCs from the identical cell line in our previously published literature were used to evaluate the cytocompatibility of the recombinant spider silk-coated film [27]. NSCs proliferated on the coated film and a significant difference in cell viability was observed between Day 1 and Day 3. The proliferation rates of NSCs were similar to that of NSCs on other biocompatible films [27]. Based on the results, the cytocompatibility of the recombinant spider silk-coated film was confirmed. Overall, the main goal of this study focuses on the self-healing mechanism of the recombinant spider silk hydrogel and surface coating. Although the surface of the coated and dried recombinant spider silk was rough, a smoother film may be fabricated by drying the hydrogel directly in the desired container after dialysis, instead of moving it to other containers before drying.

Natural dragline spider silk has some fascinating properties when it is wetted. One of the properties is known as supercontraction, which is the ability of the silk to shrink significantly in response to changes in humidity [2]. This property plays an important role in the functional adaptation of spider webs. Under high relative humidity, the spider silk in the supercontracted state behaves as a rubber network because the hydrogen bonding within the amorphous region of the spider silk is broken [64,65]. Meanwhile, the electrical conductivity of the spider silk at this state was found to be enhanced, compared to that of the original dry sample [66]. The conductivity ranges from 10^−4^ mS/m to 10^0^ mS/m, depending on the relative humidity. The enhanced conductivity of the spider silk under hydration could be attributed to the increased mobility of the silk protein chains [67,68]. Furthermore, it is worth mentioning that water can be retained in the silk even after subsequent drying [69]. In this study, the dried recombinant spider silk film fabricated from the hydrogel showed a conductivity of ~0.4 mS/m, which is compatible with the previous literature. In addition, the thin recombinant spider silk coating with cytocompatibility and biodegradability has great potential for biomedical applications, such as protecting various surfaces of medical implants [70]. Overall, we expect that the self-healing property and the healing mechanism of the recombinant spider silk hydrogel and cast thin films from the hydrogel in this study will assist in the design and optimization of self-healing silk-related biomaterials for fundamental research and functional applications. The potential limitation of the fabricated recombinant spider silk hydrogel in this study was the relatively low G′ (~250 Pa). For our future work, we plan to develop a series of recombinant spider silk hydrogels with tunable mechanical properties for broader biomedical applications. Furthermore, recombinant spider silk hydrogels incorporating different biomaterials will be developed to achieve multi-intelligent hydrogels with various functionalities (e.g., 3D printable self-healing hydrogel).

## 5. Conclusions

The self-healing recombinant spider silk hydrogel and thin coating were fabricated. The soft self-healing recombinant spider silk hydrogel (~250 Pa) demonstrated a high strain-sensitive property (critical strain ~50%), which could be ascribed to the supramolecular interactions of the hydrogen bonding. The possible self-healing mechanism examined by in situ SAXS was mainly based on the stick-slip behavior of the β-sheet nanocrystals. Through the rupture and reformation of the reversible hydrogen bonding within the β-sheet nanocrystals, the self-healing behavior of the recombinant spider silk hydrogel was achieved. For the cast and dried coating, the possible self-healing mechanism was based on the chain diffusion within the amorphous region and the reversible hydrogen bonding within the β-sheet nanocrystals. Furthermore, the coated substrate demonstrated cytocompatibility (~2.3-fold increase in the proliferation of NSCs after 3 days) and an electrical conductivity of ~0.4 mS/m. The developed biomimetic self-healing recombinant spider silk hydrogel, coating, and dried films may have good potential for broad biomedical applications.

## Data Availability

Not applicable.
